# Spheroid Culture of Mesenchymal Stromal Cells Results in Morphorheological Properties Appropriate for Improved Microcirculation

**DOI:** 10.1002/advs.201802104

**Published:** 2019-02-19

**Authors:** Stefanie Tietze, Martin Kräter, Angela Jacobi, Anna Taubenberger, Maik Herbig, Rebekka Wehner, Marc Schmitz, Oliver Otto, Catrin List, Berna Kaya, Manja Wobus, Martin Bornhäuser, Jochen Guck

**Affiliations:** ^1^ Biotechnology Center Center for Molecular and Cellular Bioengineering TU Dresden Tatzberg 47‐49 01307 Dresden Germany; ^2^ Max Planck Institute for the Science of Light & Max‐Planck‐Zentrum für Physik und Medizin Staudtstraße 2 91058 Erlangen Germany; ^3^ Institute of Immunology Medical Faculty Carl Gustav Carus TU Dresden Fetscherstraße 74 01307 Dresden Germany; ^4^ Medical Clinic I University Hospital Carl Gustav Carus TU Dresden Fetscherstraße 74 01307 Dresden Germany

**Keywords:** cell mechanics, mesenchymal stromal cells, mesenspheres, microcirculation mimetics

## Abstract

Human bone marrow mesenchymal stromal cells (MSCs) are used in clinical trials for the treatment of systemic inflammatory diseases due to their regenerative and immunomodulatory properties. However, intravenous administration of MSCs is hampered by cell trapping within the pulmonary capillary networks. Here, it is hypothesized that traditional 2D plastic‐adherent cell expansion fails to result in appropriate morphorheological properties required for successful cell circulation. To address this issue, a method to culture MSCs in nonadherent 3D spheroids (mesenspheres) is adapted. The biological properties of mesensphere‐cultured MSCs remain identical to conventional 2D cultures. However, morphorheological analyses reveal a smaller size and lower stiffness of mesensphere‐derived MSCs compared to plastic‐adherent MSCs, measured using real‐time deformability cytometry and atomic force microscopy. These properties result in an increased ability to pass through microconstrictions in an ex vivo microcirculation assay. This ability is confirmed in vivo by comparison of cell accumulation in various organ capillary networks after intravenous injection of both types of MSCs in mouse. The findings generally identify cellular morphorheological properties as attractive targets for improving microcirculation and specifically suggest mesensphere culture as a promising approach for optimized MSC‐based therapies.

## 1 Introduction

Mesenchymal stromal cells (MSCs) are a population of multipotent cells in almost all tissues, characterized by their unique morphology, fibroblast colony‐forming unit (CFU‐F) capacity, multilineage differentiation potential,^1^, ^2^ and immunomodulatory properties.^3^ They suppress alloantigen‐induced T cell proliferation,^4^ modulate terminal B cell differentiation,^5^ and inhibit the functions of natural killer cells^6^ and dendritic cells^7^ via the secretion of soluble factors^8^, ^9^ and direct cell–cell interactions.^10^ Owing to their immunosuppressive effects and tissue regenerative potential, MSCs have been used in clinical trials testing therapies for various diseases including graft‐versus‐host disease (GvHD) after allogenic stem cell transplantation,^11^, ^12^ autoimmune diseases,^13^ liver diseases,^14^ orthopedic injuries,^15^ cardiovascular diseases,^16^ and cancer.^17^ For these applications, large numbers of MSCs are required. Thus, they are commonly isolated from tissues of healthy human donors including bone marrow (BM) explants and expanded on rigid plastic surfaces as adherent monolayer (2D) cultures. This process is associated with depletion of less adherent earlier progenitors.^18^ Moreover, recent work has shown that long‐term culture on rigid and flat substrates fails to resemble the natural 3D bone marrow microenvironment with its unique tissue architecture and mechanical properties. This nonphysiological environment leads to reduced growth rates, loss of multipotency, and cellular senescence.^19^, ^20^ The most eminent disadvantages, however, are alterations in cytoskeletal organization and morphorheological phenotype, such as cell size and stiffness.^21^ Intravenous infusion of such nonphysiologically expanded MSCs results in cell trapping within the pulmonary microcirculation and inefficient homing to target organs in mice^22^, ^23^ and humans.^24^

The morphological and mechanical properties of cells have moved into the focus of attention since recent work identified them as key regulators of cell migration,^25^ immune response,^26^ and cell polarization.^27^ Changes in cell deformability, especially cell softening, not only accompany cell differentiation^28^ and motility, but also improve cell passage through microcapillaries.^29^ This raises the question whether engineering MSCs with circulation‐appropriate morphorheological properties can improve microcirculation. As a cell's morphology and mechanical phenotype depend strongly on environmental physical cues and the resulting extracellular to intracellular signaling,^30^ we asked whether the modulation of morphorheological features can be achieved using innovative cell culture techniques. Expansion of MSCs in 3D spheroidal aggregates compared to traditional 2D techniques had resulted in cells with smaller size^31^, ^32^ and reduced Young's modulus.^33^

Therefore, we adapted a scaffold‐free 3D cell culture system for the monoclonal expansion of MSCs in nonadherent spheroids—so‐called mesenspheres. We verified that 3D‐expanded cells fulfill properties of MSCs including self‐renewability, multilineage differentiation, and immune modulation. Based on our findings of cytoskeletal rearrangement in 3D‐expanded MSCs, we compared morphorheological properties of MSCs after scaffold‐free 3D and traditional 2D expansion. Using atomic force microscopy (AFM) indentation measurement and real‐time deformability cytometry (RT‐DC), we found that 3D‐expanded MSCs were smaller and more compliant. Ex vivo microcirculation analysis revealed that mesensphere‐forming single cells required less time to enter and pass a microfluidic microcirculation mimetic (MMM) device compared to plastic‐adherent MSCs. Furthermore, we demonstrated that after intravenous injection of MSCs in the tail vein of mice, mesensphere‐derived MSCs were detected in larger amount in the microcirculation of various organs other than the lungs. In this respect, the unique morphorheological properties of mesensphere‐expanded MSCs make them amenable for the development of advantageous stem cell therapies to overcome limitations that arise with traditional 2D cultures.

## 2 Results

### 2.1 Mesenspheres Comprise Multipotent, Self‐Renewable, and Immunomodulatory MSCs Capable of Hematopoietic Stem and Progenitor Cell Support

**Figure 1:**
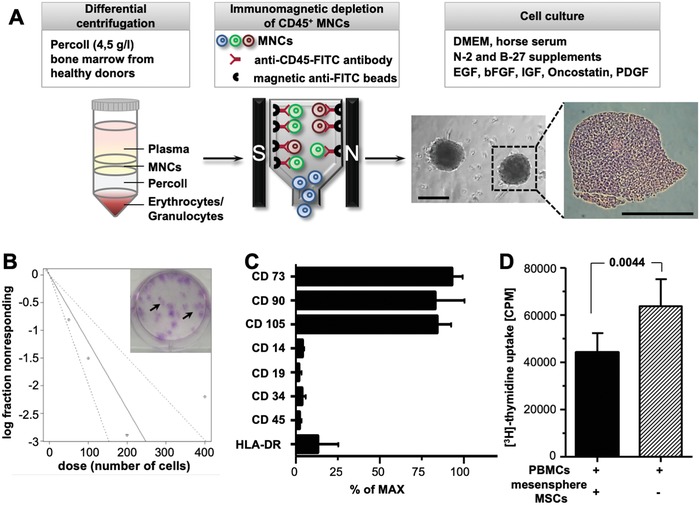
Mesenspheres comprise multipotent, self‐renewable, and immunomodulatory MSCs. A) Cell culture of mesenspheres, MSCs were isolated from bone marrow of healthy donors by density centrifugation and immunomagnetic depletion of CD45‐positive mononuclear cells (MNCs). Representative hematoxylin and eosin (H&E) staining of paraffin‐embedded mesensphere sections. Scale bar, 200 µm. B) Extreme limiting dilution assay (ELDA) of mesensphere MSCs, after a two‐week culture period mesensphere MSCs were plated at a density of 5, 10, 20, and 40 cells cm^−2^. The frequency of mesenchymal progenitors was calculated using ELDA method by counting fibroblast colonies in each dilution of three independent experiments including three technical replicates. Representative picture showing colony‐forming unit fibroblast (CFU‐F) assay using mesensphere MSCs at a clonal density of 40 cells cm^−2^. C) Flow cytometry analyses of mesensphere MSCs for minimal criteria cell surface marker expression (CD73^+^, CD90^+^, CD105^+^, CD14^−^, CD19^−^, CD34^−^, CD45^−^, HLA‐DR^−^). Histogram bars representing mean ± s.e.m. of four independent replicates. D) Modified mixed lymphocyte reaction assay. The histogram represents [3H]‐thymidine incorporation into CD3/CD28‐stimulated peripheral blood mononuclear cells (PBMCs) after coculture with irradiated (30 Gy) mesensphere MSCs (1/30 (PBMC/MSC) ratio). Histogram bars represent mean ± s.d. of five independent experiments. Statistical significance was determined using an unpaired two‐tailed *t*‐test.

Mesenspheres were cultured from BM mononuclear cell (MNC) fraction isolated by density gradient centrifugation and immunomagnetic depletion of CD45‐positive cells. Utilizing ultralow attachment flasks MSCs formed self‐assembled spherical‐shaped mesenspheres. Hematoxylin and eosin (H&E) staining of paraffin‐embedded sections revealed that mesenspheres build a compact 3D network composed of small round cells surrounded by elongated flat cells (**Figure** **1**A). To determine the frequency of mesenchymal progenitors, we performed CFU‐F assays. Mesensphere‐derived MSCs were found to establish multiple fibroblast colonies of various sizes. Using limiting dilution analysis (ELDA)^34^ the estimate of stem cell frequency in mesenspheres was 1/82.7 cells with 95% confidence interval of 1/50.9 to 1/134 cells (Figure 1B). According to the minimal criteria for MSCs,^35^ mesensphere cells were found positive for surface marker expression of CD105 (85.5% ± 8.5%), CD73 (94.3% ± 5.7%), and CD90 (85% ± 16%) and lack expression of CD14 (4.6% ± 1.2%), CD19 (2.2% ± 2%), CD34 (2.5% ± 1.6%), CD45 (4.2% ± 5%), and HLA‐DR (13.4% ± 12.4%; Figure 1C). Moreover, we found that mesenspheres exhibit multilineage differentiation potential (Figure S1A–E, Supporting Information). Immunomodulatory properties of mesensphere‐derived MSCs were investigated using a modified mixed lymphocyte reaction assay.^36^ Lymphocyte proliferation as measured by [3H]‐thymidine incorporation was significantly decreased in the presence of mesensphere MSCs (44 097.7 cpm ± 23 200.0 vs 63 718.3 cpm ± 32 461.1; *p* = 0.0044; Figure 1D). Furthermore, BM‐derived MSCs have been shown to support hematopoietic stem and progenitor cell (HSPC) maintenance and engraftment.^37^ We confirmed HSPC expansion with clonogenic and appropriate differentiation potential in coculture with mesenspheres (Figure S2A–G, Supporting Information).

Together, these results show that the biological properties of mesensphere‐derived MSCs are identical to those cultured conventionally on rigid 2D plastic surfaces. However, after intravenous administration of such expanded MSCs cell delivery to target tissues is limited as a result of cell trapping within the pulmonary capillary networks.^23^

### 2.2 Mesensphere‐Derived MSCs Exhibit Characteristic Morphorheological Properties

**Figure 2:**
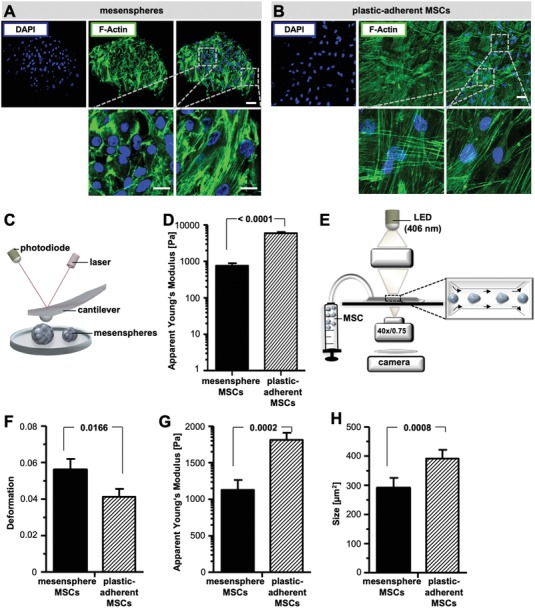
Mesensphere‐derived MSCs exhibit exceptional morphorheological properties. A,B) Representative confocal microscopy maximal projections (*Z* = 15 µm) of mesensphere cytoskeletal structures and plastic‐adherent MSC cytoskeletal structures via staining of filamentous actin (F‐actin, green) and nuclei (blue). Scale bars: upper panels, 65 µm; lower panels, 15 µm. C) Schematic presentation of atomic force microscopy (AFM) measurement of cell stiffness by indentation at mesenspheres and plastic‐adherent MSCs. The mechanical properties of the cells were quantified using the apparent Young's modulus calculated from the obtained force–indentation curves. D) Histogram bars represent mean ± s.e.m. of two independent atomic force microscopy measurements including nine technical repeats. Statistical significance was determined using an unpaired two‐tailed *t*‐test. E) In real‐time deformability cytometry (RT‐DC), mesensphere MSCs and plastic‐adherent MSCs were flowed through a 30 µm × 30 µm microfluidic constriction and deformed without contact by shear stress and pressure gradients. F–H) Cellular morphorheological properties of mesensphere MSCs and plastic‐adherent MSCs were quantified as cell deformation, apparent Young's modulus, and cell size (cross‐sectional area; µm^2^) using automated image analysis. Histogram bars represent mean ± s.d. of four independent experiments. Statistical significance was determined using a 1D linear mixed model and a likelihood ratio test.

It has been shown that the size and the amount of cytoskeletal proteins of mesenspheres are drastically reduced compared to 2D cultured MSCs.^31^, ^38^ Therefore, we studied whether MSCs cultured in mesenspheres show other relevant morphorheological properties that can influence microcirculation. Analysis of the cytoskeleton distribution in entire mesenspheres revealed short, tightly bundled actin filaments in the apical mesensphere region. Toward the mesensphere interior, actin filaments assembled as cell cortex associated ring‐like belt structures (**Figure** **2**A). In contrary, in plastic‐adherent MSCs, we observed uniformly distributed actin stress fibers, namely, dorsal stress fibers, transverse arcs, and ventral stress fibers (Figure 2B). Since cytoskeletal mutations directly correlate with morphorheological properties,^29^, ^39^, ^40^ we performed AFM indentation measurements on single MSCs in entire mesenspheres and MSCs cultured on rigid plastic surfaces to probe cell stiffness (Figure 2C). The cell mechanical properties were quantified by extracting the apparent Young's modulus from the obtained force–indentation curves. As demonstrated in Figure 2D, apparent Young's moduli measured for mesenspheres were significantly lower compared to plastic‐adherent MSCs (761.4 Pa ± 542.4 vs 5926 Pa ± 486.9, *p* = 4.9 × 10^−8^). Interestingly, the apparent Young's moduli of mesensphere cells that had been migrated out of mesenspheres after 5 h were increased compared to cells within the spheres (1871 Pa ± 971.1 vs 761.4 Pa ± 542.4, *p* = 0.001), which identifies plastic adherence as a main contributor to cell mechanics. Furthermore, morphorheological properties of suspended MSCs were analyzed using RT‐DC (Figure 2E). Here, the apparent Young's modulus can be derived from image analysis that quantified cell size and the resulting deformation as cells pass through a narrow microfluidic channel, similar to blood flow, in real time and with high throughput (up to 1000 cells s^−1^).^39^, ^41^, ^42^ In comparison with plastic‐adherent MSCs, mesensphere‐derived MSCs appeared significantly smaller (cross‐sectional area: 290.5 µm^2^ ± 34.9 vs 391.02 µm^2^ ± 30.4, *p* = 0.0008; Figure 2F) and more deformable (deformation: 0.056 ± 0.006 vs 0.041 ± 0.004, *p* = 0.0166, apparent Young's modulus: 1129.6 ± 135.0 Pa vs 1812.6 Pa ± 98.0, *p* = 0.0002; Figure 2G,H). So far, it has been assumed that the large size of MSCs cultured on rigid 2D plastic surfaces causes trapping within the pulmonary capillaries.^23^ However, our previous studies indicate that in addition to cell size, also the cell mechanical properties affect microcirculation.^29^ Therefore, we hypothesized that altered morphorheological properties of MSCs cultivated in mesenspheres can improve microcirculation.

### 2.3 Morphorheological Properties of Mesensphere‐Derived MSCs Permit Cell Circulation

**Figure 3:**
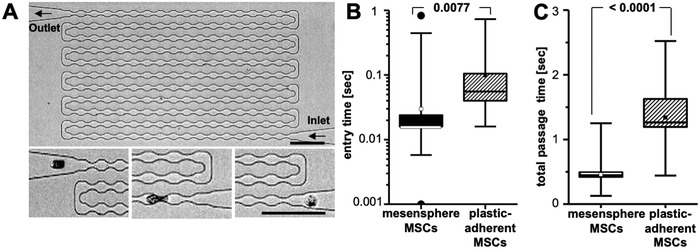
Ex vivo microfluidic microcirculation mimetic analysis of mesensphere‐derived and plastic‐adherent MSCs. A) Phase contrast image of an MMM with 187 successive constrictions (5 µm in width). Smaller inlets show the close‐up of a cell before the first constriction, after the first two constrictions, and after the last constriction (from right to left). Scale bars are 100 µm. B) Box & whiskers blots represent entry time (passage through the first constriction) and C) total passage time (from inlet to outlet) for mesensphere MSCs and plastic‐adherent MSCs to enter and pass the MMM. Lines represent mean ± s.d. of two independent experiments including 30 technical repeats. Statistical analyses were determined using an unpaired two‐tailed *t*‐test.

To analyze the impact of the culture system, and the resulting altered morphorheological properties, on MSC circulation, we mimicked pulmonary microcirculation in MMM. This microfluidic device consists of a serpentine microchannel 300 µm in total length and 15 µm in height and 15 µm in width with 187 successive constrictions of 5 µm in width (**Figure** **3**A).^43^, ^44^ These constrictions are smaller than the cell sizes tested and emulate the diameters of constrictions found in the microcapillary bed of the lung, which range from 2 to 15 µm. Thus, the deformations of the cells are caused by tactile squeezing through the constrictions and occur sequentially as in the microcirculation. Cells were resuspended in phosphate buffered saline (PBS) and flushed through the MMM using a constant pressure difference of 150 mbar between the inlet and the outlet. This pressure difference is even higher than the typical pulmonary capillary pressure of around 20 mbar. To quantify the ability of cells to pass through the constrictions, we measured entry time (time that is needed to pass the first constriction of the microchannel) and total passage time. Mesensphere MSCs were found to require less time to enter the first constriction (0.035 s ± 0.0127 vs 0.106 s ± 0.0261, *p* = 0.0077) and less time to pass the whole microchannel (0.470 s ± 0.0167 vs 1.384 s ± 0.0778, *p* < 0.0001) compared to plastic‐adherent MSCs (Figure 3B,C). We therefore concluded that, in addition to cell morphology, also physical deformability affects microcirculation of MSCs. Interestingly, we found that 2D expanded MSCs rapidly change their morphorheological phenotype when cells were detached and grown in nonadherent 96‐well plates. Here, cells stick together and form spheroidal aggregates (secondary spheres) within 1 d (15 000 cells per sphere). After dissociation of secondary spheres, we obtained MSCs with morphorheological properties (deformation and size) similar to those expanded in mesenspheres (Figure S3A–C, Supporting Information). Moreover, these cells compared to plastic‐adherent MSCs were also characterized by improved circulation through MMM with 8 µm in width (Figure S3D, Supporting Information). These findings confirm that the morphorheological phenotype of MSCs can be manipulated by the appropriate cell culture technique.

**Figure 4:**
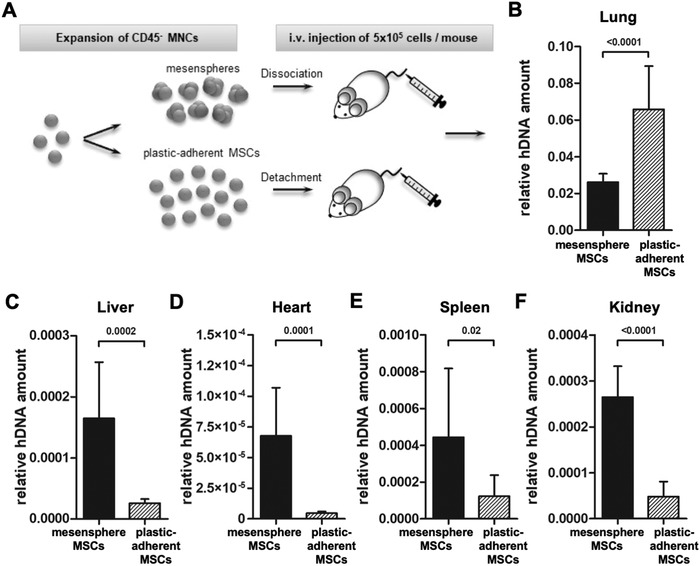
Microcirculatory properties of mesensphere and plastic‐adherent expanded MSCs. A) For analysis of the microcirculatory properties, 5 × 10^5^ MSCs from mesenspheres or 2D cultures were injected intravenously into the tail vein of NSG mice. Human MSCs were detected via amplification of human Alu sequences 15 min after i.v. infusion. B–F) Histogram bars represent the relative human DNA amount normalized to the total glyceraldehyde‐3‐phosphate‐dehydrogenase GAPDH amount (2^−ΔCT^) in lung, liver, heart, spleen, and kidney 15 min after i.v. infusion of mesensphere MSCs in comparison to plastic‐adherent MSCs. Histogram bars represent mean ± s.d. of 12 independent experiments. Statistical significance was determined using an unpaired two‐tailed *t*‐test.

To further investigate whether the unique morphorheological properties of mesensphere‐derived MSCs promote increased microcirculation, MSCs derived from mesenspheres or plastic‐adherent cultures were dissociated to obtain single‐cell suspensions and injected intravenously into the tail vein of NSG mice (**Figure** **4**A). After 15 min mice were sacrificed, organs were dissected, and DNA was isolated to quantify human Alu sequences within tissues. As depicted in Figure 4B, real‐time PCR for human Alu sequences revealed that mesensphere MSCs can pass lung capillaries more efficiently resulting in a more even distribution within the capillary networks of other tissues. Compared to 2D cultured MSCs, lung trapping of mesensphere MSCs decreased by about 30%, whereas recovery in liver, heart, spleen, and kidney increased up to 20% (Figure 4C–F). These findings demonstrate that efficient microcirculation of MSCs is determined by their morphorheological phenotype. Moreover, this microcirculation appropriate morphorheological phenotype can be engineered using 3D mesensphere instead of 2D plastic‐adherent culture techniques and thus might be crucial for effective future MSC‐based therapies.

## 3 Discussion and Conclusion

Since Friedenstein et al. described MSCs as a clonogenic population of BM‐derived cells,^45^ they have been applied in numerous clinical trials due to their regenerative and immunomodulatory potential. However, prior to the use of MSCs as cell‐based therapeutics, considerable hurdles must be overcome such as the establishment of standards for cell purification and expansion.^46^ These standards include conventional 2D monolayer cultures on rigid tissue culture plastic, which has been linked to loss of MSC function.^19^, ^20^, ^21^ Therefore, it is crucial to develop physiologically relevant culture environments that maintain MSCs in a more naïve state.

Méndez‐Ferrer et al. adapted a culture regime, conducive for the assembly of Nestin^+^ cells into neurospheroids, for Nestin^+^ murine and human BM MSCs to obtain mesenspheres.^47^ Using slightly modified culture conditions, we succeeded to obtain mesenspheres derived from CD45^−^ depleted BM‐MNC. It is supposed that spheroid generation on low attachment plates starts with loose cell aggregates mediated by extracellular matrix–integrin interactions followed by cell compaction via cadherin bindings.^38^, ^48^ The scaffold‐free culture of mesenspheres yielded a highly heterogeneous cell population in cell size and cell morphology. Irrespectively, mesensphere forming cells behave functionally like MSCs as shown by their CFU‐F capacity, cell surface molecule expression, multilineage differentiation, and HSPC support. Previous studies reported that cell expansion in 3D spheroids even enhances the differentiation potential of multipotent cells.^49^ Moreover, it is presumed that the 3D cell culture environment improves the therapeutic potential of MSCs by increased expression of relevant genes such as CXCR4 that promotes adhesion to endothelial cells^50^ or SCF that is involved in HSPC maintenance.^51^ Our analysis demonstrated that mesenspheres express high levels of HSPC‐supporting factors and are capable to expand a population containing CD45^+^/CD34^+^/CD133^+^ long‐term HSPC. Hence, efficient expansion of functional MSCs does not require plastic adherence. MSCs expansion can be achieved using scaffold‐free 3D cell culture techniques that mimic the physiological BM microenvironment more closely than plastic‐adherent 2D monolayer cultures.

Since MSCs can modulate multiple components of the immune system including T‐ and B‐cell proliferation, they emerge as promising candidates for cell‐based immunotherapies. Our analysis revealed that mesensphere forming cells effectively suppress T cell proliferation in the same manner as shown for clinical used MSCs grown in 2D monolayers.^4^ This suggests that mesensphere MSCs possess immunomodulatory properties that qualify them as therapeutics for systemic inflammatory diseases such as GvHD after allogenic hematopoietic stem cell transplantation.

So far, the most convenient route of administration for traditional 2D‐expanded MSCs is intravenous injection. Undesirably, homing to target organs is often impeded by cells trapped within the pulmonary microcapillary networks.^22^, ^23^, ^24^ Previous studies with neutrophils revealed that stiff primed compared to soft resting neutrophils exhibit longer total passage times in microcirculatory mimetics.^44^ Moreover, recent clinical observations provide evidence that primed neutrophils are retained within the pulmonary microcirculation, while deprimed neutrophils are released into the systemic circulation.^52^ Lautenschläger et al. found that the mobility of neutrophils is facilitated by cell softening, which appeared to be regulated by the actin cytoskeleton.^25^, ^29^ This implies that cell deformability must be crucial for efficient passage kinetics in microcirculation. Notably, mesensphere compared to 2D cultured MSCs appeared smaller and more deformable. These results agree with previous studies in which plastic‐adherent MSCs are described as a little‐deformable population after expansion.^53^ The altered morphorheological properties of mesensphere MSCs are even prominent in suspension after enzymatic dissociation of mesenspheres. These results may suggest that mesensphere MSCs exhibit improved microcirculatory properties. Our assumption was confirmed by ex vivo microcirculation analyses, in which mesensphere forming cells compared to plastic‐adherent MSCs required less time to enter and pass MMM. This is in line with findings by Chan et al. that showed a clear correlation between cell stiffening and extended total passage time in human promyelocytic leukemia cells (HL60). Moreover, they found that inhibition of actin polymerization with cytochalasin D decreases total passage time,^43^ which indicates that increased actin polymerization in plastic‐adherent MSCs compared to mesensphere cultured cells modulates cell stiffness and therefore might increase total passage time. The results obtained by MMM analyses were corroborated by in vivo experiments, where mesensphere MSC‐derived DNA was detected to a larger amount in organ microcirculation other than the lung capillaries after intravenous injection into the tail vein of NSG mice. The differences became particularly evident in the liver, heart, spleen, and kidney. These findings presumably predict an increased homing of MSCs to target organs. In combination with the reported immunomodulatory properties of mesensphere MSCs, these findings will be of considerable clinical significance for the treatment of systemic inflammatory diseases.

Thus, in future it might be promising to use nonadherent 3D reconstitution techniques for the development of MSC‐based therapeutic approaches in order to ensure the expansion of MSCs with small size and enhanced deformability compared to plastic‐adherent MSCs. This will prevent them from getting retained in the lung—the first major organ with an extensive microcirculatory network that MSCs have to pass—and contribute to a more even distribution of MSCs in organ capillary networks after intravenous injection. Therefore, using innovative cell culture techniques modulating the morphological and rheological phenotype of MSCs will significantly increase the feasibility of clinical stem cell therapeutic approaches.

## 4 Experimental Section

*Cell Isolation and Culture*: MSCs were isolated from healthy volunteer donors after obtaining informed written consent (Ethical Approval No. EK221102004, EK47022007) according to modifications of a previously reported method.^54^, ^55^ Briefly, human bone marrow aspirates were diluted in PBS (Thermo Fisher Scientific, Waltham, MA) at a ratio of 1:5. A 20 mL aliquot was layered over a 1073 g mL^−1^ Percoll solution (Biochrom, Berlin, Germany) and centrifuged at 2000×*g* for 15 min at room temperature. MNC fraction was recovered, washed twice in PBS, and stained with anti‐CD45‐FITC antibodies. For immunomagnetic enrichment, anti‐FITC magnetic beads (Miltenyi Biotec GmbH, Bergisch Gladbach, Germany) were used according to the manufacturer's instructions.

For clonal sphere formation, 5 × 10^6^ cells were seeded in ultralow attachment culture flasks (Greiner, Kremsmuenster, Austria). The mesensphere growth medium contained 15% horse serum, 2% B27 and 1% N2 supplements, epidermal growth factor (EGF), fibroblast growth factor‐basic (bFGF), insulin‐like growth factor (IGF; 20 ng mL^−1^), oncostatin, and platelet‐derived growth factor (PDGF; 10 ng mL^−1^) in Dulbecco's modified Eagle medium (DMEM)/F12, HEPES/human endothelial‐SFM (Thermo Fisher Scientific, Waltham, MA). The cultures were kept at 37 °C with 5% CO_2_ in a water‐jacketed incubator. For secondary sphere formation, plastic‐adherent MSCs were detached and 15 000 cells were seeded per well of a 96‐well plate precoated with 15 µL 1% agarose in DMEM + 10% FCS. Medium changes were performed weekly. For further experiments, mesenspheres were enzymatically dissociated in DMEM, high glucose supplemented with 2.2% GlutaMAX (Thermo Fisher Scientific, Waltham, MA), 0.2% type I collagenase (Wako Chemicals GmbH, Neuss, Germany), and 2.5% type I DNase (Sigma‐Aldrich, St. Louis, MO).

Mobilized peripheral blood was purchased from healthy donors treated with granulocyte colony‐stimulating factor (G‐CSF; 7.5 µg kg^−1^) for 5 d after obtaining informed written consent (Ethical Approval No. EK221102004, EK47022007). CD34^+^ HSPCs were purified from leukapheresis using anti‐CD34 magnetic beads (Miltenyi Biotec GmbH, Bergisch Gladbach, Germany) according to the manufacturer's instructions. Up to 2 × 10^4^ CD34^+^ HSPCs were cocultured with mesenspheres in low‐cytokine HSPC growth medium on agarose‐coated 24‐well plates at 37 °C with 5% CO_2_ in a water‐jacketed incubator. Low‐cytokine HSPC growth medium contained CellGro (CellGenix GmbH, Freiburg, Germany) supplemented with interleukine‐3 (IL‐3), stem cell factor (SCF), and FMS‐like tyrosine kinase‐3 (Flt‐3; 2.5 ng mL^−1^; Miltenyi Biotec GmbH, Bergisch Gladbach, Germany) on agarose‐coated 24‐well plates at 37 °C with 5% CO_2_ in a water‐jacketed incubator.

*In Vitro Differentiation*: Osteogenesis and adipogenesis were induced by culturing MSCs with the respective differentiation medium for 14 d, as described previously.^56^ Briefly, osteogenic differentiation medium contained 10 × 10^−3^ m β‐glycerophosphate, 1.5 × 10^−6^ m dexamethasone (Sigma‐Aldrich, St. Louis, MO), 0.2 × 10^−3^ m l‐ascorbate (Synopharm GmbH, Barsbuettel, Germany), and 10% FCS in DMEM (Thermo Fisher Scientific, Waltham, MA). Adipogenic differentiation was induced with 1 × 10^−9^ m dexamethasone, 500 × 10^−9^ m 3‐isobutyl‐1‐methylxanthine, 100 nm indomethacin, 1 µg mL^−1^ insulin (Sigma‐Aldrich, St. Louis, MO), and 10% FCS in DMEM. All cultures were kept at 37 °C with 5% CO_2_ in a water‐jacketed incubator. Medium changes were performed weekly.

To quantify osteogenic differentiation potential alkaline phosphatase (ALP) activity was measured as described previously.^57^ Briefly, cells were lysed in 1.5 × 10^−3^ m Tris, pH 10, containing 1 × 10^−3^ m ZnCl_2_, 1 × 10^−3^ m MgCl_2_, and 1% Triton X‐100. Lysates were clarified by centrifugation and aliquots incubated with 3.7 × 10^−3^ m 4‐nitrophenylphosphate in 100 × 10^−3^ m diethanolamine, pH 9.8, containing 0.1% Triton X‐100 for 30 min at room temperature. The reaction was stopped with 100 × 10^−3^ m NaOH and the release of 4‐nitrophenolate measured photometrically at 405 nm. In alignment with the protein concentration, ALP activity was calculated using a standard curve prepared with *p*‐nitrophenol.

For histological visualization of mesenchymal lineages, cells were washed with PBS and fixed with 4% paraformaldehyde (Merck KGaA, Darmstadt, Germany) in PBS. Cells differentiated toward osteogenic lineage were detected by van Kossa staining. Briefly, fixed cultures were stained with 1% silver nitrate (Sigma‐Aldrich, St. Louis, MO), developed using 0.1% pyrogallol and stabilized by 0.5% sodiumthiosulfate (Merck KGaA, Darmstadt, Germany). Adipogenic differentiation was assessed by Oil Red O staining. Concisely, fixed cells were stained with 0.1% Oil Red O solution (Sigma‐Aldrich, St. Louis, MO), followed by washes with distilled water.

*CFU‐F Assay*: Mononuclear cells were seeded in NH Expansion Medium (Miltenyi Biotec GmbH, Bergisch Gladbach, Germany) at a surface area density of 4 × 10^4^ cells cm^−2^. To determine the frequency of mesenchymal progenitors in mesenspheres, serial dilutions of mesensphere‐derived MSCs ranging from 50 to 400 cells in NH Expansion Medium were plated in six‐well plates. The cultures were kept at 37 °C with 5% CO_2_ in a water‐jacketed incubator. At day 14, cells were stained with Giemsa staining solution (Merck KGaA, Darmstadt, Germany) and adherent colonies were counted.

*Colony‐Forming Unit Granulocyte/Erythrocyte/Monocyte/Megakaryocyte (CFU‐GEMM) Assay*: Up to 5 × 10^2^ CD34^+^ HSPCs were cultivated in 1 mL MethoCult GF + H4435 (StemCell Technologies, Vancouver, Canada) for 14 d at 37 °C in a water‐jacketed incubator containing 5% CO_2_. The number of colony‐forming units (erythroid burst‐forming unit (BFU‐E), colony‐forming unit granulocyte (CFU‐G), colony‐forming unit monocyte (CFU‐M), colony‐forming unit granulocyte/monocyte (CFU‐GM), and CFU‐GEMM) were enumerated by optical and morphological properties using light microscopy (Axiovert 25 microscope, Carl Zeiss AG, Oberkochen, Germany).

*Flow Cytometry*: The following antibodies were used: anti‐CD45‐PECy7 (BD, Franklin Lakes, NJ), anti‐CD90‐APC (eBioscience Inc., San Diego, CA), anti‐CD105‐APC, anti‐CD34‐PE, anti‐HLA‐DR‐APC, anti‐CD73‐PE, anti‐CD14‐APC, anti‐CD19‐APC, and anti‐CD133‐FITC (Miltenyi Biotec GmbH, Bergisch Gladbach, Germany). To evaluate proliferation and apoptosis, carboxyfluorescein diacetate succinimidyl ester (CFSE) staining (Thermofisher Scientific, Waltham, MA) and propidium iodide (PI) staining (eBioscience Inc., San Diego, CA) were performed, respectively, according to the protocols of the manufacturers. Multiparameter stained cell analyses were performed using MACSQuant (Miltenyi Biotec GmbH, Bergisch Gladbach, Germany) and analyzed by FlowJo software version 7.6.5 (TreeStar Inc., Ashland, OR).

*Immunomodulatory Assay*: For immunomodulatory assay, a modified mixed lymphocyte reaction (MLR) was performed as described previously.^36^ Briefly, peripheral blood mononuclear cells (PBMCs) were isolated from healthy volunteer donors after obtaining written consent (Ethical Approval No. EK206082008). Peripheral blood samples were diluted in PBS at a ratio of 1:2. A 20 mL aliquot was layered over a 1.077 g mL^−1^ Biocoll solution (Biochrom, Berlin, Germany) and centrifuged at 1500×*g* for 30 min. MNC fraction was recovered, washed twice with PBS, and resuspended in RPMI 1640 supplemented with 10% FCS (Thermo Fisher Scientific, Waltham, MA). MLR was performed by mixing 1 × 10^4^ PBMCs, CD3/CD28 Dynabeads (Life Technologies, Carlsbad, CA) and 3 × 10^3^ irradiated (30 Gy, Gammacell 3000 Elan device, Best Theratronics Ltd., Ottawa, Canada) mesensphere MSCs or plastic‐adherent MSCs. After 4 h incubation at 37 °C with 5% CO_2_ in a water‐jacketed incubator, 1 µCi [3H]‐thymidine (Hartmann Analytic, Braunschweig, Germany) was added to the culture. After 18 h, cells were harvested by using the Inotech Cell Harvester (Inotech Biosystems International Inc., Derwood, MD). 3[H]‐thymidine incorporation was determined with the 1450 MicroBeta TriLux (PerkinElmer Inc., Waltham, MA), converting degree of radioactivity to counts per minute (cpm).

*Immunostaining and Histology*: For confocal laser scanning microscopy, mesenspheres or plastic‐adherent MSCs were fixed with 4% paraformaldehyde in PBS for 20 min. Fixed cells were stained with DAPI (Sigma Aldrich, St. Louis, MO) and phalloidin‐AF488 (Molecular Probes, Eugene, OR) according to the protocols of the manufacturers. Subsequently, specimens were coverslipped in a drop of mounting medium (Polysciences Inc., Warrington, FL) and examined by a confocal laser scanning microscope (LSM 510 Meta, Leica, Wetzlar, Germany). For histological analysis, mesenspheres were embedded in paraffin for sectioning. Microtome sections were processed with routine H&E staining and images were acquired using Axiovert 25 microscope (Carl Zeiss AG, Oberkochen, Germany).

*Atomic Force Microscopy*: Mesenspheres or 10^4^ single plastic‐adherent MSCs in CO_2_‐independent medium (Life Technologies, Carlsbad, CA) were plated into a round (diameter 35 mm) cell‐culture dish (FluoroDish, WPI, Sarasota, FL) that had been coated with 5 µg cm^−2^ fibronectin (Roche, Germany). AFM measurements were performed on a Nanowizard 4 (JPK Instruments, Berlin, Germany) equipped with a Petridishheater at 37 °C in a heat‐controlled chamber. Arrow‐T1 cantilevers (Nanoworld, Neuchatel, Switzerland) were modified with a polystyrene bead (radius 5 µm, microparticles GmbH, Berlin, Germany) to obtain a well‐defined indenter geometry and decrease local strain during indentation. Prior to the experiments, cantilevers were calibrated using built‐in procedures of the SPM software (JPK Instruments, Berlin, Germany). The cantilever/bead was positioned over single mesenspheres or plastic‐adherent MSCs, lowered at a defined speed (5 µm s^−1^) until a setpoint of 2.5 nN was reached and retracted. During the force–distance cycle, the force was recorded for each piezo position. The resulting force–distance curves were transformed into force‐versus‐tip sample separation curves and fitted with the Hertz/Sneddon model for a spherical indenter^58^, ^59^ using the JPK analysis software (JPK DP, JPK Instruments, Berlin, Germany). A Poisson ratio of 0.5 was assumed for the calculation of the apparent Young's modulus.

*Real‐Time Deformability Cytometry*: RT‐DC measurements were performed as described previously.^39^ Briefly, single cells were resuspended in PBS containing 0.63% methylcellulose (Sigma Aldrich, St. Louis, MO) at a concentration of 10^6^ cells mL^−1^. The cell suspension was drawn into a syringe and connected to a microfluidic chip containing two reservoirs connected by a 300 µm long channel (constriction) with a 30 µm × 30 µm cross section. The microfluidic chip was made of poly(dimethylsiloxane) (PDMS; Sylgard 184, VWR, Darmstadt, Germany) at which the bottom was sealed with a glass slide (Hecht, Sondheim, Germany). Using a syringe pump, cells were driven through the constriction at a constant flow rate of 0.405 µL s^−1^. Images were acquired at the end of the constriction using a CMOS camera (MC1362, Mikrotron, Unterschleissheim, Germany) which was connected to an inverted microscope (Axiovert, Carl Zeiss AG, Oberkochen, Germany). In real time, cell cross‐sectional area (size; µm^2^) and deformation were computed and plotted against each other. It has to be emphasized that deformation and size are not independent parameters for RT‐DC. This implies that for two cells of identical mechanical properties, the larger cell will deform more. A numerical model combining Stokes fluid dynamics with linear elasticity allows to disentangle the relationship of size and deformation and to deduce elastic properties, namely, Young's modulus (Mokbel. 2007) Statistical analyses were carried out using 1D linear mixed model that incorporates fixed effect parameters and random effects to analyze differences between cell subsets and replicate variances, respectively. *p*‐values were determined by a likelihood ratio test, comparing the full model with a model lacking the fixed effect term.

*Microfluidic Microcirculation Mimetic*: MMM was used, apart from minor modifications, as described previously.^43^ In short, MMM is based on a microfluidic device produced using PDMS and standard photolithographic techniques.^29^ The microfluidic chip contained an inlet and an outlet connected by a single channel (constriction) with 5 µm or 8 µm smallest in width (maximum width: 15 µm). For MMM measurements, single cells were resuspended to a final density of 3 × 10^4^ cells mL^−1^ in PBS containing 0.1% pluronic acid F‐127 (Molecular Probes Inc., Eugene, OR). Up to 50 µL of cell suspension was filled into a custom‐cut pipette tip (Eppendorf, Hamburg, Germany) and connected to the inlet region of the microfluidic chip. Using a computerized air pressure control system (MFCS‐FLEX; Fluigent, Villejuif, France) connected to the inlet and outlet of the device cells were driven through the constriction at a constant pressure of 150 mbar. The microfluidic chip was mounted on the platform of an inverted microscope, which was connected to a camera (The Imaging Source, Bremen, Germany) to record videos at a frame‐rate of 120 frames s^−1^. The first constriction entry time and the total passage time were extracted from the videos using custom‐made written codes in MATLAB (The MathWorks, Natick, MA).

*Animal Experiments*: Animal experiments were strictly performed in compliance with the animal experiment permission no. DD24.1‐5131/394/14, approved by the Landesdirektion Sachsen. A total of 1 × 10^6^ mesensphere MSCs or plastic‐adherent MSCs were intravenously injected into the tail vein of NOD scid gamma mice (NOD.Cg‐Prkdcscid Il2rgtm1Wjl/SzJ). After 15 min, organs were resected and used for DNA isolation.

*RNA Isolation, Complementary DNA Synthesis, DNA Isolation, and Real‐Time Quantitative PCR*: Total RNA was isolated by TRIzol Reagent (Ambion GmbH, Kaufungen, Germany) and transcribed into complementary DNA using RevertAid First Strand cDNA Synthesis Kit (Thermo Fisher Scientific, Waltham, MA). Real‐time quantitative PCR was performed with the primers listed in Table S1 (Supporting Information) and SYBR Green Master Mix (Thermo Fisher Scientific, Waltham, MA) in a Taqman 7000 Fast cycler (Applied Biosystems, Forster City, CA).

DNA was isolated using DNeasy Blood & Tissue Kit (Qiagen, Hilden, Germany) according to the protocols of manufacturers. Real‐time quantitative PCR for Alu sequences was performed as described previously.^60^ Briefly, 25 µL Taqman PCR Universal Master Mix (Applied Biosystems, Forster City, CA), 900 × 10^−9^ m each of the forward and reverse primers, 250 × 10^−9^ m TaqMan probe (Table S1, Supporting Information), and 200 ng target template were incubated at 50 °C for 2 min and at 95 °C for 10 min followed by 40 cycles at 95 °C for 15 s and 60 °C for 1 min.

All real‐time PCR assays were performed in duplicates. The obtained Ct values for human DNA were related to the total DNA level of an internal reference gene (glyceraldehyde 3‐phosphate dehydrogenase (GAPDH) to yield ∆CT = Ct^GAPDH^ − Ct^human Alu sequence^. The relative human DNA amount (hDNA) was calculated using the formula hDNA = 2^−∆Ct^.

*Statistical Analyses*: Despite RT‐DC data all statistical analyses were performed using GraphPad Prism Version 5.0 (San Diego, CA, USA).

## Conflict of Interest

Oliver Otto is cofounder and shareholder of Zellmechanik Dresden GmbH, a company selling real‐time deformability cytometry devices. The authors affirm that there are no other conflicts of interest (either financial or personal).

## Supporting information

Supplementary
