# Supplementary material for: Spheroid Culture of Mesenchymal Stromal Cells Results in Morphorheological Properties Appropriate for Improved Microcirculation
Source: Adv Sci (Weinh). 2019 Feb 19;6(8):1802104. doi: 10.1002/advs.201802104 (PMC6469243; doi:10.1002/advs.201802104)
Supplement: Supplementary file 1 — Supplementary [file ADVS-6-1802104-s001.pdf]

Copyright WILEY-VCH Verlag GmbH & Co. KGaA, 69469 Weinheim, Germany, 2019.

ADVANCED SCIENCE
Open Access

Supporting Information

for *Adv. Sci.,* DOI: 10.1002/advs.201802104

Spheroid Culture of Mesenchymal Stromal Cells Results in Morphorheological Properties Appropriate for Improved Microcirculation

Stefanie Tietze, Martin Kräter, Angela Jacobi, Anna Taubenberger, Maik Herbig, Rebekka Wehner, Marc Schmitz, Oliver Otto, Catrin List, Berna Kaya, Manja Wobus, Martin Bornhäuser, and Jochen Guck**

Copyright WILEY-VCH Verlag GmbH & Co. KGaA, 69469 Weinheim, Germany, 2019.

Supporting Information

Authors
Stefanie Tietze#, Martin Kräter#, Angela Jacobi, Anna Taubenberger, Maik Herbig, Rebekka Wehner, Marc Schmitz, Oliver Otto, Catrin List, Berna Kaya, Manja Wobus, Martin Bornhäuser*, and Jochen Guck*

Title
Spheroid Culture of Mesenchymal Stromal Cells Results in Morpho-Rheological Properties Appropriate for Improved Microcirculation

Supplementary Figures and Tables

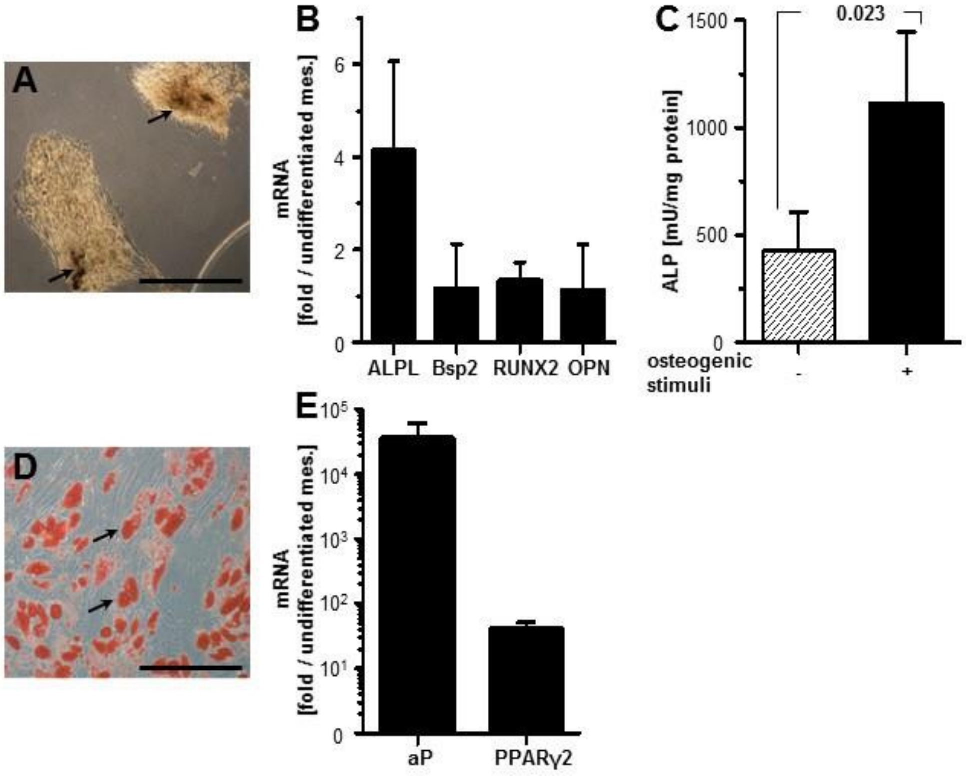

Figure S1. Multilineage differentiation potential of mesenspheres. Mesensphere MSCs were maintained in differentiation medium for 14 days. Osteogenic differentiation (A) and adipogenic differentiation (D) was revealed by van Kossa staining of calcium deposits and Oil-Red staining of lipid droplets, respectively. Scale bars, 200 µm. Expression level of osteocyte marker genes (B; alkaline phosphatase (ALPL), bone sialoprotein2 (Bsp2), runt-related transcription factor 2 (RUNX2), osteopontin (OPN)) and adipocyte marker genes (E; adipocyte protein (aP), peroxisome proliferator-activated receptor gamma (PPARγ2)) in mesensphere cells was quantified using real-time PCR in respect to control cells. Histogram bars represent mean ± s.e.m. of four independent experiments including two technical replicates. (C) Histogram display alkaline phosphatase (ALP) activity of mesensphere cells in

response to osteogenic stimuli. Histogram bars represent mean ± s.e.m. of seven independent experiments. Statistical significance was determined by an unpaired two-tailed t-test.

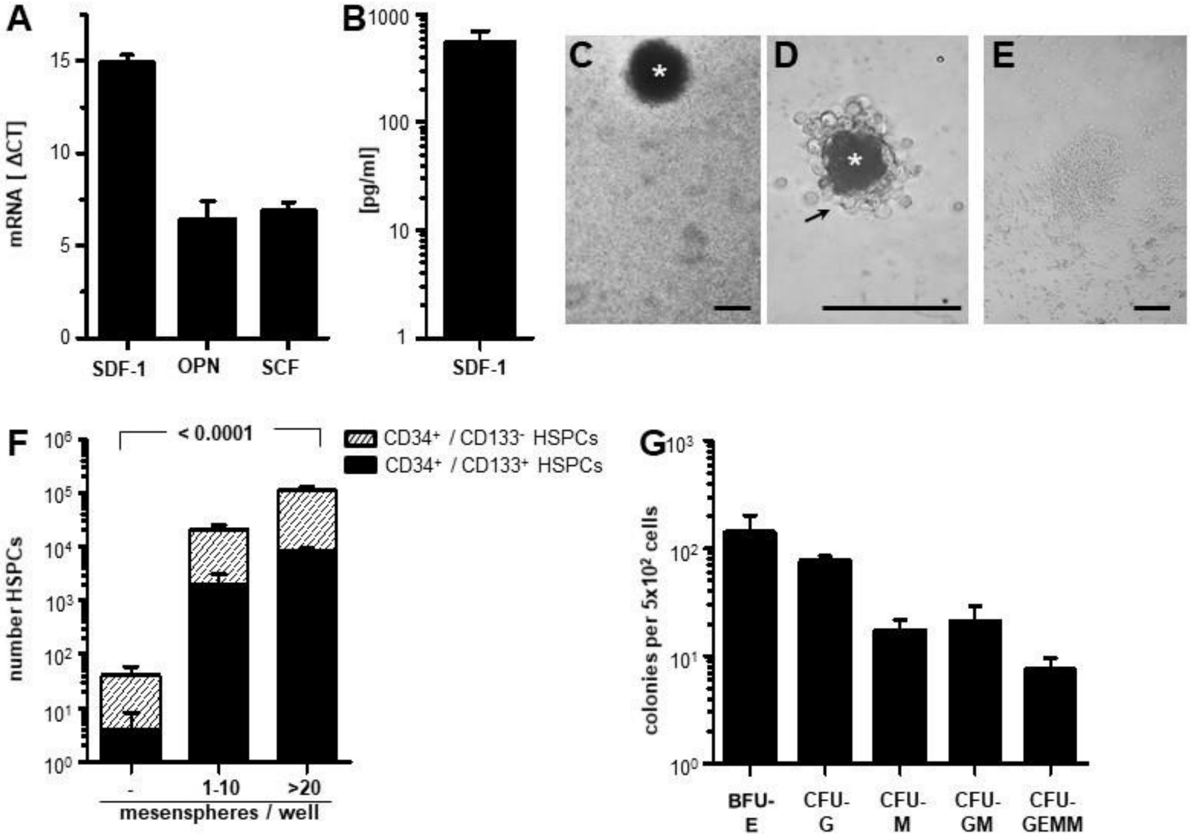

Figure S2. Ex vivo expansion of hematopoietic stem and progenitor cells in co-culture with mesenspheres. (A) Histogram show mRNA expression level of HSPC maintenance genes (stromal-derived factor 1 (SDF-1), osteopontin (OPN), stem cell factor (SCF)) in mesensphere cells normalized to glyceraldehyde-3-phosphate-dehydrogenase (GAPDH). Histogram bars represent mean ± s.d. of four independent experiments including two technical replicates. (B) SDF-1 level in cell culture supernatants of mesenspheres measured by ELISA. Histogram bars represent mean ± s.d. of six independent experiments. (C) Representative pictures show hematopoietic stem and progenitor cells (HSPCs) after 10 days in co-culture with mesenspheres (asterisk). HSPCs were detected free-floating in the supernatant or (D) directly attached to a mesensphere (asterisk). Scale bars, 200 µm (E) Representative picture showing HSPC proliferation in the absence of mesenspheres. (F) Flow cytometry analyses to determine absolute counts of CD45 and CD34 double-positive ($CD45^+$ / $CD34^+$) cells and CD45, CD34 and CD133 triple-positive ($CD45^+$ / $CD34^+$ / $CD133^+$) cells after 10 days in co-culture with mesenspheres. Histogram bars represent mean ± s.d. of two independent experiments including two technical replicates. Statistical significance was determined using a One-way ANOVA test. (G) CFU-GEMM assay of 5 x 10^2 ex vivo expanded HSPCs. Histogram bars represent mean ± s.d. of two independent experiments including three technical replicates.

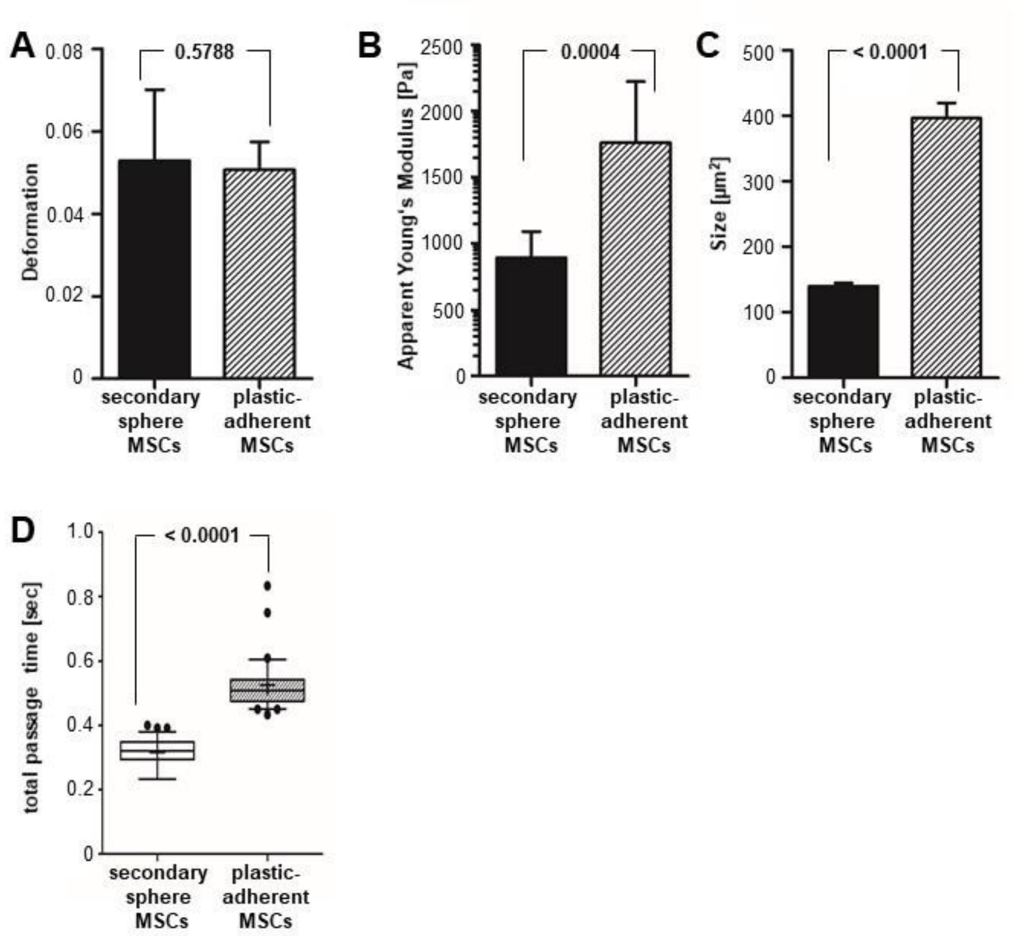

Figure S3. Morpho-rheological properties and ex vivo microcirculation of secondary sphere MSCs compared to plastic-adherent MSCs. (A-C) After RT-DC measurements, the expected cellular mechanical properties of secondary sphere MSCs and plastic-adherent MSCs were derived from image algorithm analysis that quantified (A) cell deformation, (B) apparent Young's Modulus, and (C) cell size. Histogram bars representing mean ± s.d. of four independent experiments. Statistical significance was determined using 1-dimensional linear mixed model and a likelihood ratio test. (D) Box & whiskers blots representing total passage time (from inlet to outlet) for secondary sphere MSCs and plastic-adherent MSCs to pass an MMM with 8 µm constrictions in width. Lines representing mean ± s.d. of four independent experiments including twenty technical repeats. Statistical significance was determined using an unpaired two-tailed t-test.

Table S1. PCR Primer Sequences

Primer name	Sequences (5'-3')
ALPL Forward	GAC CCT TGA CCC CCA CAA T
ALPL Reverse	GCT CGT ACT GCA TGT CCC CT
Alu Forward	CAT GGT GAA ACC CCG TCT CTA
Alu Reverse	GCC TCA GCC TCC CGA GTA G
Alu Probe	FAM- ATT AGC CGG GCG TGG TGG CG - TAMRA
aP Forward	GCA GCC TTC TCA GCC AAA C

aP Reverse	CAG CAT CTG GGT ATT TGT TGT A
Bsp2 Forward	ACC ACACTT TCT GCT ACA AC
Bsp2 Reverse	CCT CTT CCT CCT CTT CTT CTT C
hGAPDH Forward	GAA GGT GAA GGT CGG AGT C
hGAPDH Reverse	GAA GAT GGT GAT GGG ATT TC
HPRT2 Forward	TTG CGA CCT TGA CCA TCT TTG
HPRT2 Reverse	CTT TGC TGA CCT GCT GGA TTA C
m/hGAPDH Forward	AAC GAC CCC TTC ATT GAC CT
m/hGAPDH Reverse	CTC CTG GAA GAT GGT GAT GG
OPN Forward	ACAGCCAGGACTCCATTGAC
OPN Reverse	ACACTATCACCTCGGCCATC
PPARγ2 Forward	TCT CCA GCA TTT CTA CTC CAC
PPARγ2 Reverse	GGC TCC ACT TTG ATT GCA
RUNX2 Forward	GCC TTC AAG GTG GTA GCC C
RUNX2 Reverse	CGT TAC CCG CCA TGA CAG TA
SCF Forward	CTC CAG TAA GTG GCC TTT GC
SCF Reverse	TAT CTG AGG GCC TGA ACA CC
SDF-1 Forward	GGT CCG TCC TGT CTT GAT GT
SDF-1 Reverse	ACT GGG TGT ACC ACC TGC TC
